# The Importance of Patient-Reported Outcome Measures (PROMs) in Oncological Vulvoperineal Defect Reconstruction: A Systematic Review

**DOI:** 10.3390/curroncol31100470

**Published:** 2024-10-18

**Authors:** Nicole E. Speck, Julia Stoffel, Séverin Wendelspiess, Christian Appenzeller-Herzog, Kristin M. Schaefer, Loraine P. Kouba, Florian Rüter, Céline Montavon, Viola Heinzelmann-Schwarz, Martin D. Haug, Dirk J. Schaefer, Tarek Ismail, Elisabeth A. Kappos

**Affiliations:** 1 Department of Plastic, Reconstructive, Aesthetic and Hand Surgery, University Hospital Basel, 4031 Basel, Switzerland; julia.stoffel@stud.unibas.ch (J.S.); severin.wendelspiess@unibas.ch (S.W.); kristinmarit.schaefer@usb.ch (K.M.S.); lorainepascale.kouba@usb.ch (L.P.K.); martin.haug@usb.ch (M.D.H.); dirk.schaefer@usb.ch (D.J.S.); tarek.ismail@usb.ch (T.I.); elisabeth.kappos@usb.ch (E.A.K.); 2 University Medical Library, University of Basel, 4031 Basel, Switzerland; christian.appenzeller@unibas.ch; 3 Quality Management & Value Based Healthcare, University Hospital Basel, 4031 Basel, Switzerland; florian.rueter@usb.ch; 4 Department of Gynecology and Gynecological Oncology, Hospital for Women, University Hospital Basel, 4031 Basel, Switzerland; celine.montavon@usb.ch (C.M.); viola.heinzelmann@usb.ch (V.H.-S.); 5 Faculty of Medicine, University of Basel, 4031 Basel, Switzerland

**Keywords:** oncological reconstruction, vulvoperineal reconstruction, quality of life, patient-reported outcome measures, surveys and questionnaires, outcome assessment, health care

## Abstract

Background: Patient-reported outcome measures (PROMs) have gained increased importance in assessing outcomes after reconstructive surgery. This also applies to the reconstruction of vulvoperineal defects after resection of gynecological or colorectal cancers in women. The objective of this study is to analyze the current state of PROM tool use within this patient population. Methods: By systematic literature searches in Embase, Medline, and Web of Science, English-language studies published after 1980, including randomized controlled trials, cohort studies, and case series reporting on vulvoperineal defect reconstruction, which were included if they also analyzed quality of life (QoL) and/or PROMs. The PROM tools used by each study were extracted, analyzed, and compared. Results: The primary search yielded 2576 abstracts, of which 395 articles were retrieved in full text. Of these, 50 reported on vulvoperineal defect reconstruction, among which 27 studies analyzing QoL were found. Of those, 17 met the inclusion criteria for this systematic review. After full-text screening, 14 different PROM tools and 5 individual, non-standardized questionnaires were identified. Only 22% of studies used a validated PROM tool. Conclusion: Far too few studies currently use PROM tools to assess outcomes in oncological vulvoperineal defect reconstruction. Less than half of the used PROMs are validated. No PROM was designed to specifically measure QoL in this patient population. The standardized implementation of a validated PROM tool in the clinical treatment of this patient population is an essential step to improve outcomes, enable the comparison of research, and support evidence-based treatment approaches.

## 1. Introduction

Advances in cancer screening and personalized oncological therapies have led to a reduction in mortality for many patients. As such, cancer survivorship has been brought to the forefront of research [1]. Quality of life (QoL) lies at the core of cancer survivorship care. It is well known that reconstructive surgery can greatly improve the QoL of patients with oncologic defects in several anatomic regions.

Historically, defects resulting from the resection of cancerous vulvoperineal lesions have been closed primarily or left open to secondary healing for prolonged periods of time, often leading to sexual and psychosocial burdens [2]. Secondary reconstructive surgery has been reserved for patients with prolonged healing or long-term complications such as fistulas [3]. The exact percentage of women undergoing vulvoperineal defect reconstruction is unknown but may range from 9% to 71%, depending on the institution [4,5,6,7]. However, studies have shown that reconstruction may be associated with improved QoL. A prospective longitudinal study showed that patients who received a neovagina reported better physical and sexual quality of life scores than those who had no reconstruction [8]. Another study analyzing QoL after perineal flap reconstruction demonstrated that reconstruction after extensive perineal resection did not impair QoL when compared with resection without reconstruction, even though the resection size was much larger in the reconstructed group [9]. This highlights the potential role of reconstructive surgery to treat complex defects in this field.

Patient-reported outcome measures (PROMs) can be used to assess the impact of treatments, enabling a quantifiable, objective evaluation, and means of comparison [10]. Evidence shows that the routine use of PROMs in an oncological setting improves patient-provider communication and patient satisfaction [11,12]. More specifically, the field of breast reconstruction has successfully demonstrated the importance of postoncological reconstruction for an integrated treatment approach and improvement of patients’ long-term QoL [13,14,15]. The efforts in research and patient advocacy of the past decades have led to breast reconstruction being the standard of care in most centers worldwide. Concurrently, PROM research has been able to emphasize the value of these reconstructions [16,17,18]. The same cannot be said for the vulvoperineal region [19]. This anatomical region is still stigmatized and the awareness of reconstructive options within this region holds great potential. In many centers, vulvoperineal reconstruction is not offered to patients as a standard pillar of care. Cooperation between clinical specialties is often lacking, as was previously the case with breast cancer. However, collaborative efforts with breast surgeons have led to the establishment of breast centers, where patients receive care in a multidisciplinary setting from the point of diagnosis to reconstruction and follow-up care [20]. At the same time, the number of patients being offered and choosing breast cancer reconstruction has increased [21]. The same interdisciplinary treatment algorithms should be strived for in women undergoing vulvoperineal defect reconstruction after gynecologic or colorectal cancers. Analogously, if more women were offered reconstructive surgical options, this might lead to more patients electing reconstruction in this sensitive anatomical area. 

Postoncological reconstruction is arguably equally important for cancers of the vulvoperineal region as it is for breast cancer due to the functional and psychosexual importance of the vulvoperineum [22,23]. The female perineal functions comprise micturition, defecation, and reproduction, thereby contributing to QoL [24]. The perineum is also closely linked to the body image and sexual well-being [25]. The functional and psychological importance therefore mandate a thorough evaluation of the outcomes of reconstructive procedures using PROMs that can capture the diverse subjective and objective facets of the surgery. However, as we have learned from experiences in breast cancer surgery, it is indispensable to establish a standardized use of a validated PROM, such as the BREAST-Q as the clinical standard [26]. 

The aim of this systematic review is to provide an overview of the PROMs currently being used to assess QoL outcomes in women undergoing oncological vulvoperineal defect reconstruction. 

## 2. Materials and Methods

**Figure 1:**
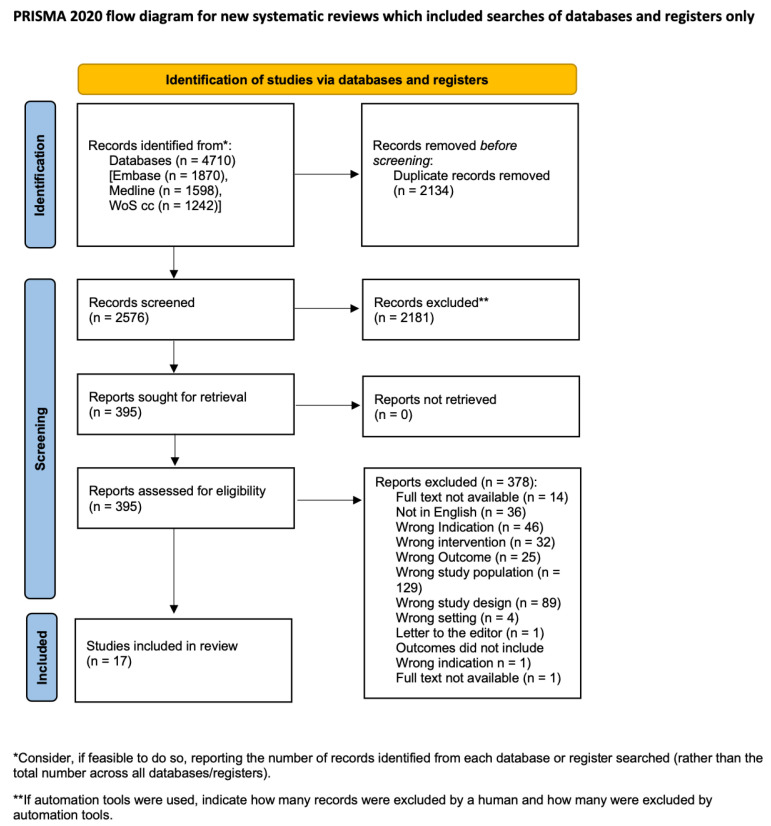
PRISMA flow diagram.

This work has been reported in line with the Preferred Reporting Items for Systematic Reviews and Meta-Analyses (PRISMA) and Assessing the methodological quality of systematic reviews (AMSTAR) Guidelines [27,28]. The PRISMA flow diagram is provided in Figure 1. This systematic review was registered in the PROSPERO International prospective register of systematic reviews (ID CRD42022384724).

### 2.1. Literature Search Strategy 

A systematic literature search was performed to identify articles on vulvoperineal defect reconstruction after cancer resections. The search strategy was developed by a medical information specialist (C.A-H.). The bibliographic databases Embase (Elsevier), Medline (Ovid), and Web of Science Core Collection (Clarivate) were searched using database-specific subject headings and text words covering the concepts pelvic cancers, pelvic reconstruction surgery, and surgical flaps (last search 28 September 2022). No language or publication date restrictions were applied but conference abstracts were excluded from the search. The full search strategies can be found in the Supplementary Materials (File S1).

### 2.2. Eligibility Criteria 

Three reviewers (J.S., S.W., and L.P.K.) independently screened all the references resulting from the search. Studies reporting on female patients undergoing vulvoperineal defect reconstruction with a perforator or non-perforator flap were included. The following flaps were considered: Singapore flap, vertical rectus abdominis muscle (VRAM), gracilis muscle, pedicled anterolateral thigh (ALT), pedicled profunda artery perforator (PAP), pedicled inferior gluteal artery perforator (IGAP) flap, thigh flaps, lotus petal flap, and gluteus myocutaneous rotation flap. Non-English studies and studies with primary defect closure were excluded. Any articles mentioning QoL, health-related QoL, or PROMs were then screened in full text by two independent reviewers (J.S. and N.E.S.) for the following additional eligibility criteria: (1) adults; (2) PROM tool or other questionnaire for female patients; (3) defect due to oncologic tumor resection. Studies that did not assess QoL or did not use the flaps of interest were excluded. Disagreements were solved by consensus or an additional reviewer (E.A.K.).

### 2.3. Assessment of Methodological and Evidence Quality

Two reviewers (J.S. and N.E.S.) used the Cochrane Risk of Bias in Non-Randomized Studies—of Interventions (ROBINS—I) tool to independently assess the risk of bias in the included observational studies.

### 2.4. Data Extraction 

The full-text articles to be screened for this study were reviewed using a data-extraction sheet created on Covidence.org with the following predetermined variables: study ID, title, authors and year of publication, country in which the study was conducted, study population (number of participants, age, oncological disease or other etiology), start and end date, (neo-)adjuvant chemotherapy or radiation therapy, surgical approach or flap type, surgical indication, and PROM tool used. The complete data-extraction sheet can be found in File S2. As the goal of this study was to provide an overview of the PROM tools currently being used, the PROMs cited by each study were extracted and evidence of their development and validation was obtained. A qualitative assessment of the different studies using a standardized methodological assessment tool was beyond the scope of this systematic review. 

## 3. Results

The initial electronic database search identified 2576 unique records, of which 395 were forwarded to further screening. Of these, 50 reported on vulvoperineal defect reconstruction and were selected for full-text screening. After full-text screening, 27 articles were identified that mentioned QoL or PROMs. These 27 articles were then screened in detail to identify articles that specifically used a PROM tool or an individual, non-standardized questionnaire to assess QoL after surgery. Of the 27 articles, 17 met the inclusion criteria for this systematic review [9,29,30,31,32,33,34,35,36,37,38,39,40,41,42,43,44]. A total of 471 patients were included in the analyzed studies. Within the 17 included articles, 14 different PROM tools, and five individual, non-standardized questionnaires were found. The subset of non-standardized questionnaires was summarized into one group. A list of the PROM tools used by each study and of all PROMs sorted by frequency can be found in Table 1.

**Figure 2:**
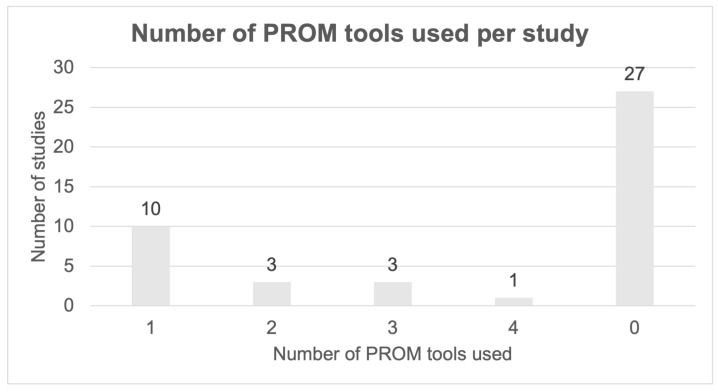
Number of PROM tools used per study.

Of the 17 articles assessing QoL after oncological vulvoperineal defect reconstruction, 6 articles used individual, non-standardized questionnaires, either in combination with a PROM tool (*n* = 2) or as their only measurement tool (*n* = 4). The number of PROM tools used per study is summarized in Figure 2. 

The PROM tools were grouped as follows: generic (*n* = 5), general oncology (*n* = 2), gynecological (*n* = 1), and PROMs relating to sexual function/pelvic floor function and incontinence or colorectal cancer (*n* = 6). File S3 provides a detailed list of the frequency of PROM tool use by group. The PROMs used in each study are shown in Table 2.

Most studies used either a generic tool (5/14 = 35.7%) or a tool relating to sexual function, pelvic floor, or incontinence (4/14 = 28.6%). The most frequently used tool was the Female Sexual Function Index (FSFI). It was used by five articles, one of which used it as their only tool and four of which used it in combination with one or more other tools. Only two articles used the same single tool, the EuroQol Group (EQ-5D), and two studies used the same combination of tools. The EQ-5D was the most frequently used tool in the “generic group”, while the European Organization for Research and Treatment of Cancer Quality of Life Questionnaire (EORTC QLQ-C30) was most used in the “general oncology group” and the FSFI in the “sexual function/pelvic floor pelvic floor function and incontinence or colorectal cancer group”.

Using the results of the initial search on vulvoperineal defect reconstruction, we were able to calculate the frequency of PROM tools used among this patient population using the following equation: (11 articles using at least one validated PROM tool)/(50 articles on oncological vulvoperineal defect reconstruction within this patient population) = a frequency of 0.22 = 22% of articles reporting on vulvoperineal defect reconstruction which used validated PROMs to assess surgical outcomes.

## 4. Discussion

### 4.1. Historical Development of PROM Tool Use Within This Patient Population

According to this systematic review, far too few studies currently use PROM tools to assess outcomes in oncological vulvoperineal defect reconstruction with less than half of the used PROMs being validated, and no PROM being designed to specifically measure QoL in this patient population. When looking at the evolution of PROM tool use within this patient population over the past 20 years, two articles from 2002 and 2022 can be used in an illustrative manner. In the article published by Mirhashemi et al. in 2002, the authors chose to have one of their residents interview their patients on the phone due to the lack of a suitable tool [29]. Twenty years later, Pai et al. still deemed it necessary to use a self-developed, non-standardized questionnaire because they were not able to find an existing tool that was suitable to answer their research question. They created their own questions focusing on QoL parameters such as ability to sit comfortably, perineal pain, and perineal aesthetics as reported by patients [32]. As stated by the authors themselves, the consequence is the limited comparability of their study. This highlights the urgency for the standardized use of a validated PROM tool for this patient population. To support this development, we examined the current state of the use of PROM instruments through this systematic review. Such reviews provide a comprehensive overview of the measurement properties of PROMs and subsequently provide an evidence-based recommendation on the selection of the most suitable PROM tool for a given population [45]. They help to identify gaps in the literature, highlighting which patient populations need the development of a new PROM tool for their individual needs and outcomes, thereby raising awareness on the issue amongst clinically practicing surgeons whilst serving as a call for action among the scientific community. 

### 4.2. Functional and Psychological Relevance of the Vulvoperineal Region

The vulvoperineal region holds a comparable significance in shaping a woman’s physical and psychosexual identity as the breast due to its multiple functional roles, including micturition, defecation, and reproduction. These roles emphasize the importance of implementing PROMs into routine clinical care, as the assessment of outcomes is highly individual to each person and can only be evaluated by the patients themselves. The long-term goal should be to reach levels of QoL like those we have achieved in oncoplastic breast reconstructive surgery. Previous studies have shown that vulvoperineal reconstruction may be associated with improved QoL. A prospective longitudinal study showed that patients who received a neovagina had better physical and sexual QoL scores than those who had no reconstruction [8]. Another study comparing QoL after perineal resection with and without reconstruction with the lotus petal flap found no significant difference between both groups [9]. However, there was a trend towards higher QoL scores in the reconstructed group. Importantly, complex flap reconstruction did not decrease QoL even though the resection size was much larger in the reconstructed group. This result highlights the role and potential of a multidisciplinary approach that includes oncologic and reconstructive surgery.

### 4.3. PROMs in Other Fields of Plastic and Reconstructive Surgery

Apart from breast cancer patients, other patient populations in plastic and reconstructive surgery have also benefited from the implementation of a standardized tool, namely the transgender population with the introduction of the GENDER-Q, released in April 2023 [46]. The validation and implementation of this tool helped to improve access to gender-equitable care [47,48]. This is an impressive demonstration of how a standardized approach to QoL assessment makes it possible to synthesize data across studies, enabling a coherent and standardized approach to the care of a patient population [10]. 

### 4.4. Heterogeneity of PROMs Used After Oncoplastic Vulvoperineal Defect Reconstruction

Our analysis showed that there are currently 14 different PROM tools, and a multitude of individual, non-standardized questionnaires, which were used by a total of 17 articles reporting on vulvoperineal defect reconstruction and QoL. None of the PROMs were developed or validated for specific use in this population. The high number of PROMs strongly suggests that there is a lack of consensus among surgeons and clinician-scientists as to which instrument is most suitable for this population. Moreover, the fact that the PROM tools stem from different clinical subspecialties (i.e., oncology, gynecology, gynecologic oncology, sexual medicine, and urology) shows that there is a need for the standardization of PROM tool use, incorporating valuable insights from all the above-listed specialties. This is highlighted by the fact that 36% of the studies deemed it necessary to develop their own individual and non-standardized questionnaire, used either in combination with an existing tool or on its own. As stated by Pusic et al., patient questionnaires that are not formally developed or tested (ad hoc questionnaires) may pose reasonable questions, but unless they are psychometrically tested, we cannot be confident about their reliability (i.e., ability to produce consistent and reproducible scores) or validity (i.e., ability to measure what is intended to be measured) [49].

Additionally, we found that several studies used a generic PROM tool. A generic instrument is a broad-based questionnaire, such as the Short Form-36, that measures health-related QoL in diverse patient populations [50]. This is concerning because research has shown that although these tools may be reliable, they are not sensitive enough to assess a patient’s specific condition [49,51,52,53]. In contrast, condition-specific PROMs have greater face validity, credibility, and responsiveness to changes in the patient’s condition, which is especially important when comparing pre- and postoperative functions [50]. Moreover, the tools stemming from the field of general oncology, such as the Functional Assessment of Cancer Therapy–General (FACT-G), are not sufficiently specific either. Many of the general oncology tools were developed with the effects of chemotherapy, radiation, and immunotherapy in mind, but do not include questions related to reconstructive surgery, such as wound healing or change in body image. Also, the tools in general do not focus on any specific anatomic region and the related symptoms, which, in our case being the pelvic region, is highly relevant [54]. A combination of a general tool and a more specific tool, however, might be advisable, as promoted in the PROMIS [55].

### 4.5. Validation of PROM Tools

Only 7 of the 14 PROM tools we identified are validated (50%). PROMs were defined as validated according to Churruca et al. if there are published statistical analyses establishing reliability (e.g., Cronbach’s alpha) and validity of the scale(s), including construct validity (e.g., factor analyses, item-response modeling, convergent and discriminant validity), criterion-related validity (e.g., concurrent and predictive validity), or analyses of known group differences. These validation analyses had to be conducted on an English-language version of the instrument, either in the original validation paper or subsequently [56]. A list of the validated tools and their respective validation studies can be found in Table 3. It is important to emphasize that although the tools are validated, none of them were developed or validated specifically for patients undergoing oncological vulvoperineal defect reconstruction. 

### 4.6. The FSFI as the Most Commonly Used Tool

The FSFI, which was the most-used tool in our analysis, is the gold standard for the measurement of sexual function in women [63]. It has undergone extensive psychometric validation via studies involving female gynecological, bone marrow, cervical, and breast cancer patients with appropriately matched controls [60,64]. This does not cover all the entities which are included in this patient population, such as vulvar cancer, anal cancer, and rectal cancer. Also, we do not know if this instrument is able to differentiate between the various types of cancer treatments received, e.g., surgery, radiation, or chemotherapy, and how these may impact the validity of the scoring system. Additionally, the FSFI only pertains to a 4-week period, meaning patients who were not sexually active in the least 4 weeks would obtain a score of “0” in this section. Depending on when the questionnaire is applied in the postoperative period, this may limit the applicability of this tool for patients who were not sexually active in the last 4 weeks after surgery. Newer tools such as the Information on Sexual Health: Your Needs after Cancer (InSYNC) questionnaire were designed specifically for cancer survivors and are therefore better equipped to inquire about the full complexity of changes in sexual health after cancer and cancer treatment. A further aspect to be considered is that some of the tools that were used may be discriminating towards certain groups of patients such as women in same-sex relationships or women with multiple sexual partners. According to Tounkel et al., a heterosexual bias was found to be present in the FSFI, the SVQ (Sexual Function and Vaginal Changes Questionnaire) and the SAQ (Sexual Activity Questionnaire). A heterosexual bias was defined as the targeting of the questions towards heterosexual practices and intercourse (penile–vaginal intercourse) [65].

### 4.7. Consequences of Heterogeneous PROM Tool Use in Clincal Care and Research

The heterogeneity of PROM tools used in women after oncoplastic vulvoperineal defect reconstruction poses a serious limitation for research on a national or international level, as patient-reported outcomes cannot be objectively compared and standardized between centers. This hinders the overarching goal of improving the surgical outcomes and QoL of patients undergoing vulvoperineal defect reconstruction [66]. Furthermore, research performed with outcome measurement instruments of poor or unknown quality constitutes a waste of resources and can even be deemed unethical, as it imposes the burden of questionnaires onto patients without providing them or future patients the possibility of benefiting from the knowledge or insights gained through the evaluation of their answers. This is contrary to the basic principles of clinical research, which include the enhancement of potential benefits for subjects and society and promoting the health of the population as a whole [45,67,68,69].

### 4.8. PROMs in the Age of Value-Based Healthcare

Value-based healthcare aims to improve the quality of care delivered by measuring and improving outcomes that reflect value rather than volume [70,71,72]. PROMs are gaining increasing recognition as a reliable method to assess the quality and effectiveness of medical interventions [73,74]. With the growing focus on value-based healthcare, there is increasing pressure on providers to deliver these data as proof of patient-centered, efficient, and high-quality treatment [75,76]. PROMs are a valuable instrument for measuring the impact of reconstructive surgeries on patients’ QoL over time and the systematic collection of standardized and validated PROMs allows the creation of large databases, providing useful long-term information for scientific and clinical purposes and further facilitating clinical decision-making and quality control. The ability to compare research results means that the findings gathered with PROMs can even be used in discussions with regulatory authorities, making them politically relevant [10,77,78]. Thus, it is of utmost importance to implement PROMs into clinical routine and research in the long term. As described by van Egdom et al., designing an outcomes set using PROMs was essential in reflecting the entire cycle of care and long-term sustainability of QoL in the implementation of a Value-Based Breast Cancer Care Center at an academic tertiary hospital [71]. A similar outcome set should be agreed upon for vulvoperineal cancer patients. Their proposed framework may act as a blueprint for the implementation of value-based health-care pathways in female cancer care [71]. This raises the question of whether a tool tailored specifically to the needs of a patient population needs to be created, which, similarly to the BREAST-Q, can reach a generalized level of acceptance and implementation amongst the scientific community. It is debatable whether this population needs a new tool or if the standardized use of one of the existing tools would be sufficient. Nevertheless, the absence of a suitable tool is a probable explanation as to why none of the current tools have been established as the gold standard in clinical routine and research. As stated by Beelen et al., the development of a validated PROM is a time-intensive process, but it is a vital step in creating an adequate instrument [66]. Substantial patient input in the form of interviews would provide content validity and enable a more appropriate measurement of the outcomes important to patients. Qualitative research is necessary to better understand the aspects of QoL and treatment outcomes most important to patients undergoing reconstructive surgery in this region [10,49]. Since patients with vulvoperineal malignancies are treated in an interdisciplinary setting, inputs from all the involved disciplines, namely gynecology, urology, urogynecology, gastroenterology and proctology, radiation oncology, and plastic and reconstructive surgery, must be considered in the development process.

### 4.9. Limitations

There are some limitations to this study. Articles that only assessed functionality and did not explicitly mention the assessment of QoL were not included in the frequency equation. Ideally, having identified a lack of standardized PROM tool use in female patients undergoing vulvoperineal defect reconstruction, our study would include a new PROM tool specifically designed for this population. However, as mentioned by Cano et al., careful and extensive qualitative work is necessary to conceptualize a new formal PROM [79]. A literature review is part of the first stage of development, which needs to be supplemented with expert opinion, ideally synthesized in an expert consensus meeting including patient advocates, alongside detailed patient interviews to design an exhaustive list of potential items for the first draft of a questionnaire. The development of the BREAST-Q and, more recently, the LYMPH-Q, serve as examples of a thorough PROM development process [80,81]. While the development of a new PROM tool was therefore beyond the scope of this systematic review, we strongly advocate the formal development of a PROM tool for female patients receiving vulvoperineal defect reconstruction and see this review as a call to action to said issue.

## 5. Conclusions

The standardized implementation of a highly validated PROM tool has been established in multiple areas of reconstructive surgery, breast reconstruction being the prime example. In contrast, this systematic review shows that there is currently no evidence for the standardized use of PROM tools in patients undergoing pelvic reconstructive surgery after gynecological or colorectal cancers. The use of PROMs within this patient population is scarce and highly fragmented, and there is no single instrument that is specific enough to capture the individual needs of this patient population and no instrument that has been established as the gold standard in clinical care and research. The overarching goal should be to reach the same level of standardized use and implementation as the BREAST-Q, and thereby establish reconstruction as a standardized pillar of care in the treatment of patients with oncologic disease of the vulvoperineum, enable the comparison of research, and support evidence-based treatment approaches.

## Figures and Tables

**Table 1:** List of all PROM tools used in a total of 17 studies (sorted by frequency) (*n* = 15).

No.	PROM Tool	No. Studies Using This PROM Tool
0	Individual, non-standardized questionnaire [1,29,31,32,33]	5
1	EORTC QLQ-C30 [1,30,40,42]	4
2	FSFI [1,30,34,39,40]	5
3	EORTC QLQ-CR29 [1,30,42]	3
4	SF-36 [36,41]	2
5	BIS [40]	1
6	FACT-G [42]	1
7	FACT-V [43]	1
8	FACT-C [41]	1
9	Cleveland Clinic QoL [42]	1
10	EQ-5D [37,38]	2
11	FIQL [36]	1
12	mSAQ [35]	1
13	SVQ [44]	1
14	MOS SF-36 [34]	1

Abbreviations: BIS, Body Image Scale; EORTC QLQ-CR29 European Organization for Research and Treatment of Cancer Quality of Life Questionnaire colorectal cancer; EORTC QLQ-C30, European Organization for Research and Treatment of Cancer Quality of Life Questionnaire; EQ-5D, EuroQol Group; FACT-C, Functional Assessment of Cancer Therapy for patients with colorectal cancer; FACT-G, Functional Assessment of Cancer Therapy–General; FACT-V, Functional Assessment of Cancer Therapy–Vulva; FIQL, fecal incontinence quality of life scale; FSFI, Female Sexual Function Index; MOS SF-36, Medical Outcome Study Short Form-36; mSAQ, Modified Sexual Adjustment Questionnaire; SF-36, Short Form-36; SVQ, Sexual function Vaginal changes Questionnaire.

**Table 2:** PROMs used by each study.

Study Title	Reference	PROM Tool Used
Vaginal reconstruction at the time of pelvic exenteration: a surgical and psychosexual analysis of techniques	Mirhashemi et al., 2002 [29]	Individual, non-standardized questionnaire
Vaginal reconstruction using a gluteal transposition flap after abdominoperineal excision for anorectal malignancy	Bolmstrand et al., 2022 [30]	EORTC QLQ-C30; EORTC QLQ-CR29; FSFI
Complications and sexual function after vaginectomy for anorectal tumors	Hendren et al., 2007 [31]	Individual, non-standardized questionnaire
Analysis of clinical and patient-reported outcomes in post-ELAPE perineal reconstruction with IGAP flap—A 5-year review	Pai et al., 2022 [32]	Individual, non-standardized questionnaire
Surgical and psychosexual outcome following vaginal reconstruction with pelvic exenteration	Gleeson et al., 1994 [33]	Individual, non-standardized questionnaire
Quality of life and female sexual function after skinning vulvectomy with split-thickness skin graft in women with vulvar intraepithelial neoplasia or vulvar Paget disease	Lavoué et al., 2013 [34]	FSFI; MOS SF-36
Sexual dysfunction after colpectomy and vaginal reconstruction with a vertical rectus abdominis myocutaneous flap	Love et al., 2013 [44]	Sexual function Vaginal changes Questionnaire
Patient-reported outcomes and sexual function in vaginal reconstruction: a 17-year review, survey, and review of the literature	Scott et al., 2010 [35]	mSAQ
Long-term functional and quality of life outcomes of patients after repair of large perianal skin defects for Paget’s and Bowen’s disease	Conklin et al., 2009 [36]	SF-36; FIQL
Pelvic exenteration for advanced and recurrent malignancy	Zoucas et al., 2012	EQ-5D
Physical performance and quality of life after extended abdominoperineal excision of rectum and reconstruction of the pelvic floor with gluteus maximus flap	Haapamäki et al., 2011 [38]	EQ-5D
Female sexual function after abdominoperineal resection for squamous cell carcinoma of the anus and the specific influence of colpectomy and vertical rectus abdominis myocutaneous flap	Corte et al., 2011 [39]	FSFI
Quality of Life and Sexual Functioning After Vulvar Reconstruction With the Lotus Petal Flap	Hellinga et al., 2018 [40]	EORTC QLQ-C30; FSFI; Body Image Scale
Complications and Impact on Quality of Life of Vertical Rectus Abdominis Myocutaneous Flaps for Reconstruction in Pelvic Exenteration Surgery	vanRamshorst et al., 2020 [41]	SF-36; FACT-C
Quality of Life, Sexual Functioning, and Physical Functioning Following Perineal Reconstruction with the Lotus Petal Flap	Hellinga et al., 2020 [9]	EORTC QLQ-C30; EORTC QLQ-CR29; FSFI; individual, non-standardized questionnaire
Vertical rectus abdominis myocutaneous flap and quality of life following abdominoperineal excision for rectal cancer: a multi-institutional study	O’Dowd et al., 2014 [42]	EORTC QLQ-C30; EORTC QLQ-CR29; Cleveland Clinic QOL
Profunda artery perforator flap for isolated vulvar defect reconstruction after oncological resection	Chang et al., 2016 [43]	FACT-V; FACT-G

**Table 3:** Validated PROM tools and their corresponding validation studies.

PROM Tool	Validation Study
EORTC QLQ-C30	Aaronson et al., 1993 [57]
FACT-G	Cella et al., 1993 [54]
EQ-5D	Janssen et al., 2013 [58]
FACT-V	Janda et al., 2005 [59]
FSFI	Baser et al., 2012 [60]
SF-36	Brazier et al., 1992 [61]
EORTC QLQ-CR29	Whistance et al., 2009 [62]

## Supplementary Materials

The following supporting information can be downloaded at: https://www.mdpi.com/article/10.3390/curroncol31100470/s1, File S1: full search strategy; File S2: data-extraction sheet; File S3: overview of the PROM tools used and their respective frequency.
