# Supplementary material for: The Importance of Patient-Reported Outcome Measures (PROMs) in Oncological Vulvoperineal Defect Reconstruction: A Systematic Review
Source: Curr Oncol. 2024 Oct 18;31(10):6300–13. doi: 10.3390/curroncol31100470 (PMC11506363; doi:10.3390/curroncol31100470)
Supplement: Supplementary file 1 [file curroncol-31-00470-s001.zip › curroncol-3246186-file S1 and S3.pdf]

Current Oncology

Supplementary Materials

The Importance of Patient-Reported Outcome Measures (PROMs) in Oncological Vulvoperineal Defect Reconstruction: A Systematic Review

File S1 Full search strategy

Embase.com

(20220928; 1870 hits)

((('female genital tract tumor'/de OR 'female genital tract cancer'/de OR 'vagina tumor'/exp OR 'vulva tumor'/exp OR 'colorectal tumor'/de OR 'colorectal cancer'/de OR 'anorectal tumor'/exp OR 'pelvis tumor'/exp OR 'perineum tumor'/de OR 'perineum cancer'/de OR 'perineum carcinoma'/de OR 'giant condyloma acuminatum'/de OR (('neoplasm'/exp OR 'cancer surgery'/de OR 'surgical oncology'/de OR 'adenomectomy'/de) AND ('vagina'/exp OR 'perineum'/exp OR 'perineum injury'/de OR 'rectum'/exp OR 'anus'/exp)) OR (((gynecologic* OR gynaecologic* OR pelvis OR pelvic OR 'female genital*' OR vagina OR vagine OR vaginal OR vulva OR vulvar OR perineum OR perineal OR abdominoperineal OR vulvoperineal OR rectum OR rectal OR recti OR mesorectum OR mesorectal OR colorectal OR rectovaginal OR anus OR anal OR anorectal OR anovaginal OR perivulvar OR perirectal OR perianal OR rectosigmoid OR 'lower sigmoid' OR 'lower abdomen' OR Buschke-Lowenstein OR Buschke-Loewenstein) NEAR/3 (cancer* OR neoplas* OR dysplas* OR tumor* OR tumour* OR carcinoma* OR adenocarcinoma OR adenoma* OR carcinogenesis OR malignan* OR melanoma OR polyp* OR pseudopolyp* OR papilloma*)) OR 'giant condyloma'):ab,ti)

AND

('gynecologic surgery'/de OR 'vagina surgery'/de OR 'vaginectomy'/exp OR 'vagina reconstruction'/de OR 'vulvectomy'/de OR 'vulva surgery'/de OR 'perineal surgery'/de OR 'perineal reconstruction'/de OR 'rectum surgery'/de OR 'rectum resection'/exp OR 'anus surgery'/de OR 'anoplasty'/de OR 'abdominoperineal excision'/de OR 'colectomy'/de OR 'sigmoidectomy'/de OR 'reconstructive surgery'/de OR 'plastic surgery'/de OR 'microvascular surgery'/exp OR 'pelvis surgery'/de OR 'pelvis exenteration'/de OR 'pelvis lymphadenectomy'/de OR 'pelvic floor reconstructive surgery'/de OR 'pelvic floor reconstruction'/de OR 'pelvic floor repair'/de OR (((gynecologic* OR gynaecologic* OR pelvis OR pelvic OR 'female genital*' OR vagina OR vagine OR vaginal OR vulva OR vulvar OR perineum OR perineal OR abdominoperineal OR vulvoperineal OR rectum OR rectal OR mesorectum OR mesorectal OR colorectal OR rectovaginal OR anus OR anal OR anorectal OR anovaginal OR perivulvar OR perirectal OR perianal OR rectosigmoid OR 'lower sigmoid' OR 'lower abdomen' OR hindquarter OR local) AND (surgery OR surgical OR repair* OR operation* OR operative OR excis* OR resect* OR amputation OR exstirpation OR extirpation OR exenteration OR lymphadenectom*)) OR colpoplast* OR vaginoplast* OR colpectom* OR colpocleisis OR vaginectom* OR vulvectom* OR anoplast* OR proctectom* OR symphysiotom* OR hemipelvectom* OR colectom* OR sigmoidectom* OR neocolpopoiesis OR 'miles operation' OR 'Brunschwig operation' OR reconstruct* OR 'plastic surg*' OR 'microvascular surg*' OR 'surgical palliation'):ab,ti))

OR

(('female genital tract tumor'/de OR 'female genital tract cancer'/de OR 'vagina tumor'/exp OR 'vulva tumor'/exp OR 'colorectal tumor'/de OR 'colorectal cancer'/de OR 'anorectal tumor'/exp OR 'pelvis tumor'/exp OR 'perineum tumor'/de OR 'perineum cancer'/de OR 'perineum carcinoma'/de OR 'giant condyloma acuminatum'/de OR (('neoplasm'/exp OR 'cancer surgery'/de OR 'surgical oncology'/de OR 'adenomectomy'/de OR (tumor* OR tumour* OR carcinoma* OR adenocarcinoma OR adenoma* OR

carcinogenesis OR melanoma OR polyp* OR pseudopolyp* OR papilloma*):ab,ti) AND ('vagina'/exp OR 'perineum'/exp OR 'perineum injury'/de OR 'rectum'/exp OR 'anus'/exp OR (gynecologic* OR gynaecologic* OR pelvis OR pelvic OR 'female genital*' OR vagina OR vagine OR vaginal OR vulva OR vulvar OR perineum OR perineal OR abdominoperineal OR vulvoperineal OR rectum OR rectal OR recti OR mesorectum OR mesorectal OR colorectal OR rectovaginal OR anus OR anal OR anorectal OR anovaginal OR perivulvar OR perirectal OR perianal OR rectosigmoid OR 'lower sigmoid' OR 'lower abdomen' OR Buschke-Lowenstein OR Buschke-Loewenstein):ab,ti)) OR ('giant condyloma' OR oncolog* OR cancer OR neoplas* OR dysplas* OR malignan*):ab,ti)

AND

('gynecologic surgery'/de OR 'vagina surgery'/de OR 'vaginectomy'/exp OR 'vagina reconstruction'/de OR 'vulvectomy'/de OR 'vulva surgery'/de OR 'perineal surgery'/de OR 'perineal reconstruction'/de OR 'rectum surgery'/de OR 'rectum resection'/exp OR 'anus surgery'/de OR 'anoplasty'/de OR 'abdominoperineal excision'/de OR 'colectomy'/de OR 'sigmoidectomy'/de OR 'pelvis surgery'/de OR 'pelvis exenteration'/de OR 'pelvis lymphadenectomy'/de OR 'pelvic floor reconstructive surgery'/de OR 'pelvic floor reconstruction'/de OR 'pelvic floor repair'/de OR (('reconstructive surgery'/de OR 'plastic surgery'/de OR 'microvascular surgery'/exp) AND ('vagina'/exp OR 'perineum'/exp OR 'perineum injury'/de OR 'rectum'/exp OR 'anus'/exp)) OR (((gynecologic* OR gynaecologic* OR pelvis OR pelvic OR 'female genital*' OR vagina OR vagine OR vaginal OR vulva OR vulvar OR perineum OR perineal OR abdominoperineal OR vulvoperineal OR rectum OR rectal OR mesorectum OR mesorectal OR colorectal OR rectovaginal OR anus OR anal OR anorectal OR anovaginal OR perivulvar OR perirectal OR perianal OR rectosigmoid OR 'lower sigmoid' OR 'lower abdomen' OR hindquarter) NEAR/5 (surgery OR surgical OR reconstruct* OR repair* OR operation* OR operative OR excis* OR resect* OR amputation OR exstirpation OR extirpation OR exenteration OR lymphadenectom*)) OR colpoplast* OR vaginoplast* OR colpectom* OR colpocleisis OR vaginectom* OR vulvectom* OR anoplast* OR proctectom* OR symphysiotom* OR hemipelvectom* OR colectom* OR sigmoidectom* OR neocolpopoiesis OR 'miles operation' OR 'Brunschwig operation'):ab,ti)))

AND

('surgical flaps'/exp OR 'tissue flap'/de OR 'adipofascial flap'/de OR 'anterolateral thigh flap'/de OR 'chimeric flap'/de OR 'deep inferior epigastric perforator flap'/de OR 'fasciocutaneous flap'/de OR 'gracilis flap'/de OR 'inferior gluteal artery perforator flap'/de OR 'inguinal flap'/de OR 'island flap'/de OR 'latissimus dorsi flap'/de OR 'muscle flap'/de OR 'myocutaneous flap'/de OR 'perforator flap'/de OR 'skin flap'/exp OR 'transverse rectus abdominis musculocutaneous flap'/de OR 'vertical rectus abdominis musculocutaneous flap'/de OR 'venous flap'/de OR 'omental flap'/de OR 'v y plasty'/de OR 'skin graft'/de OR 'full thickness skin graft'/de OR (((surgical OR free OR tissue OR adipofascial OR adipo-fascial OR 'anterolateral thigh' OR 'antero-lateral thigh' OR ALT OR 'medial thigh' OR 'posterior thigh' OR pudendal-thigh OR chimeric OR fasciocutaneous OR fascio-cutaneous OR gracilis OR IGAP OR I-GAP OR inguinal OR groin OR island OR muscle OR myocutaneous OR myo-cutaneous OR muscular-cutaneous OR musculocutaneous OR musculo-cutaneous OR perforator OR skin OR cutaneous OR dermal OR pedicle* OR bipedicle* OR transposition* OR Singapore OR VRAM OR gluteal OR gluteus OR omentum OR omental OR peritoneum OR peritoneal OR V-Y OR fillet OR local OR advancement OR 'fasciae latae' OR Martius) NEAR/3 (flap OR flaps OR graft OR grafts)) OR 'free inguinal graft' OR 'skin pedicle' OR 'flap repair' OR 'flap reconstruction*' OR 'V-Y plasty'):ab,ti)

NOT

(('animal'/de OR 'animal experiment'/exp OR 'nonhuman'/de) NOT ('human'/exp OR 'human experiment'/de))

NOT

[conference abstract]/lim

Medline (Ovid)

(20220628; Ovid MEDLINE(R) ALL 1946 to September 27, 2022; 1598 hits)

((("genital neoplasms, female"/ OR exp vaginal neoplasms/ OR exp vulvar neoplasms/ OR colorectal neoplasms/ OR exp rectal neoplasms/ OR sigmoid neoplasms/ OR pelvic neoplasms/ OR (exp neoplasms/ AND (exp vagina/ OR exp vulva/ OR perineum/ OR rectum/ OR anal canal/)) OR (((gynecologic* OR gynaecologic* OR pelvis OR pelvic OR female genital* OR vagina OR vagine OR vaginal OR vulva OR vulvar OR perineum OR perineal OR abdominoperineal OR vulvoperineal OR rectum OR rectal OR recti OR mesorectum OR mesorectal OR colorectal OR rectovaginal OR anus OR anal OR anorectal OR anovaginal OR perivulvar OR perirectal OR perianal OR rectosigmoid OR lower sigmoid OR lower abdomen OR Buschke-Lowenstein OR Buschke-Loewenstein) ADJ3 (cancer* OR neoplas* OR dysplas* OR tumor* OR tumour* OR carcinoma* OR adenocarcinoma OR adenoma* OR carcinogenesis OR malignan* OR melanoma OR polyp* OR pseudopolyp* OR papilloma*)) OR giant condyloma).ab,ti.)

AND

(gynecologic surgical procedures/ OR exp vagina/su OR exp vulva/su OR exp perineum/su OR exp rectum/su OR anal canal/su OR vulvectomy/ OR reconstructive surgical procedures/ OR surgery, plastic/ OR exp pelvis/su OR (((gynecologic* OR gynaecologic* OR pelvis OR pelvic OR female genital* OR vagina OR vagine OR vaginal OR vulva OR vulvar OR perineum OR perineal OR abdominoperineal OR vulvoperineal OR rectum OR rectal OR mesorectum OR mesorectal OR colorectal OR rectovaginal OR anus OR anal OR anorectal OR anovaginal OR perivulvar OR perirectal OR perianal OR rectosigmoid OR lower sigmoid OR lower abdomen OR hindquarter OR local) AND (surgery OR surgical OR repair* OR operation* OR operative OR excis* OR resect* OR amputation OR exstirpation OR extirpation OR exenteration OR lymphadenectom*)) OR colpoplast* OR vaginoplast* OR colpectom* OR colpocleisis OR vaginectom* OR vulvectom* OR anoplast* OR proctectom* OR symphysiotom* OR hemipelvectom* OR colectom* OR sigmoidectom* OR neocolpopoiesis OR miles operation OR Brunschwig operation OR reconstruct* OR plastic surg* OR microvascular surg* OR surgical palliation).ab,ti.))

OR

(("genital neoplasms, female"/ OR exp vaginal neoplasms/ OR exp vulvar neoplasms/ OR colorectal neoplasms/ OR exp rectal neoplasms/ OR sigmoid neoplasms/ OR pelvic neoplasms/ OR ((exp neoplasms/ OR (tumor* OR tumour* OR carcinoma* OR adenocarcinoma OR adenoma* OR carcinogenesis OR melanoma OR polyp* OR pseudopolyp* OR papilloma*).ab,ti.) AND (exp vagina/ OR exp vulva/ OR perineum/ OR rectum/ OR anal canal/ OR (gynecologic* OR gynaecologic* OR pelvis OR pelvic OR female genital* OR vagina OR vagine OR vaginal OR vulva OR vulvar OR perineum OR perineal OR abdominoperineal OR vulvoperineal OR rectum OR rectal OR recti OR mesorectum OR mesorectal OR colorectal OR rectovaginal OR anus OR anal OR anorectal OR anovaginal OR perivulvar OR perirectal OR perianal OR rectosigmoid OR lower sigmoid OR lower abdomen OR Buschke-Lowenstein OR Buschke-Loewenstein).ab,ti.)) OR (giant condyloma OR oncolog* OR cancer OR neoplas* OR dysplas* OR malignan*).ab,ti.)

AND

(gynecologic surgical procedures/ OR exp vagina/su OR exp vulva/su OR exp perineum/su OR exp rectum/su OR anal canal/su OR vulvectomy/ OR exp pelvis/su OR ((reconstructive surgical procedures/ OR surgery, plastic/) AND (exp vagina/ OR exp vulva/ OR perineum/ OR rectum/ OR anal canal/)) OR (((gynecologic* OR gynaecologic* OR pelvis OR pelvic OR female genital* OR vagina OR vagine OR vaginal OR vulva OR vulvar OR perineum OR perineal OR abdominoperineal OR vulvoperineal OR rectum OR rectal OR mesorectum OR mesorectal OR colorectal OR rectovaginal OR anus OR anal OR anorectal OR anovaginal OR perivulvar OR perirectal OR perianal OR rectosigmoid OR lower sigmoid OR lower abdomen OR hindquarter) ADJ5 (surgery OR surgical OR reconstruct* OR repair* OR operation* OR operative OR excis* OR resect* OR amputation OR exstirpation OR extirpation OR exenteration OR lymphadenectom*)) OR colpoplast* OR

vaginoplast* OR colpectom* OR colpocleisis OR vaginectom* OR vulvectom* OR anoplast* OR proctectom* OR symphysiotom* OR hemipelvectom* OR colectom* OR sigmoidectom* OR neocolpopoiesis OR miles operation OR Brunschwig operation).ab,ti.)))

AND

(exp surgical flaps/ OR (((surgical OR free OR tissue OR adipofascial OR adipo-fascial OR anterolateral thigh OR antero-lateral thigh OR ALT OR medial thigh OR posterior thigh OR pudendal-thigh OR chimeric OR fasciocutaneous OR fascio-cutaneous OR gracilis OR IGAP OR I-GAP OR inguinal OR groin OR island OR muscle OR myocutaneous OR myo-cutaneous OR muscular-cutaneous OR musculocutaneous OR musculo-cutaneous OR perforator OR skin OR cutaneous OR dermal OR pedicle* OR bipedicle* OR transposition* OR Singapore OR VRAM OR gluteal OR gluteus OR omentum OR omental OR peritoneum OR peritoneal OR V-Y OR fillet OR local OR advancement OR fasciae latae OR Martius) ADJ3 (flap OR flaps OR graft OR grafts)) OR free inguinal graft OR skin pedicle OR flap repair OR flap reconstruction* OR V-Y plasty).ab,ti.)

NOT (exp animals/ NOT humans/)

Web of Science Core Collection

(20220028; Web of Science Core Collection, Editions = A&HCI , BKCI-SSH , BKCI-S , CCR-EXPANDED , ESCI , IC , CPCI-SSH , CPCI-S , SCI-EXPANDED , SSCI; 1242 hits)

TS=((((((((gynecologic* OR gynaecologic* OR pelvis OR pelvic OR "female genital*" OR vagina OR vagine OR vaginal OR vulva OR vulvar OR perineum OR perineal OR abdominoperineal OR vulvoperineal OR rectum OR rectal OR recti OR mesorectum OR mesorectal OR colorectal OR rectovaginal OR anus OR anal OR anorectal OR anovaginal OR perivulvar OR perirectal OR perianal OR rectosigmoid OR "lower sigmoid" OR "lower abdomen" OR Buschke-Lowenstein OR Buschke-Loewenstein) NEAR/2 (cancer* OR neoplas* OR dysplas* OR tumor* OR tumour* OR carcinoma* OR adenocarcinoma OR adenoma* OR carcinogenesis OR malignan* OR melanoma OR polyp* OR pseudopolyp* OR papilloma*)) OR "giant condyloma")))

AND

((((gynecologic* OR gynaecologic* OR pelvis OR pelvic OR "female genital*" OR vagina OR vagine OR vaginal OR vulva OR vulvar OR perineum OR perineal OR abdominoperineal OR vulvoperineal OR rectum OR rectal OR mesorectum OR mesorectal OR colorectal OR rectovaginal OR anus OR anal OR anorectal OR anovaginal OR perivulvar OR perirectal OR perianal OR rectosigmoid OR "lower sigmoid" OR "lower abdomen" OR hindquarter OR local) AND (surgery OR surgical OR repair* OR operation* OR operative OR excis* OR resect* OR amputation OR exstirpation OR extirpation OR exenteration OR lymphadenectom*)) OR colpoplast* OR vaginoplast* OR colpectom* OR colpocleisis OR vaginectom* OR vulvectom* OR anoplast* OR proctectom* OR symphysiotom* OR hemipelvectom* OR colectom* OR sigmoidectom* OR neocolpopoiesis OR "miles operation" OR "Brunschwig operation" OR reconstruct* OR "plastic surg*" OR "microvascular surg*" OR "surgical palliation")))

OR

(((((tumor* OR tumour* OR carcinoma* OR adenocarcinoma OR adenoma* OR carcinogenesis OR melanoma OR polyp* OR pseudopolyp* OR papilloma*) AND (gynecologic* OR gynaecologic* OR pelvis OR pelvic OR "female genital*" OR vagina OR vagine OR vaginal OR vulva OR vulvar OR perineum OR perineal OR abdominoperineal OR vulvoperineal OR rectum OR rectal OR recti OR mesorectum OR mesorectal OR colorectal OR rectovaginal OR anus OR anal OR anorectal OR anovaginal OR perivulvar OR perirectal OR perianal OR rectosigmoid OR "lower sigmoid" OR "lower abdomen" OR Buschke-Lowenstein OR Buschke-Loewenstein)) OR "giant condyloma" OR oncolog* OR cancer OR neoplas* OR dysplas* OR malignan*))

AND

((((gynecologic* OR gynaecologic* OR pelvis OR pelvic OR "female genital*" OR vagina OR vagine OR vaginal OR vulva OR vulvar OR perineum OR perineal OR abdominoperineal OR vulvoperineal OR rectum OR rectal OR mesorectum OR mesorectal OR colorectal OR rectovaginal OR anus OR anal OR anorectal OR anovaginal OR perivulvar OR perirectal OR perianal OR rectosigmoid OR "lower sigmoid" OR "lower abdomen" OR hindquarter) NEAR/5 (surgery OR surgical OR reconstruct* OR repair* OR operation* OR operative OR excis* OR resect* OR amputation OR exstirpation OR extirpation OR exenteration OR lymphadenectom*)) OR colpoplast* OR vaginoplast* OR colpectom* OR colpocleisis OR vaginectom* OR vulvectom* OR anoplast* OR proctectom* OR symphysiotom* OR hemipelvectom* OR colectom* OR sigmoidectom* OR neocolpopoiesis OR "miles operation" OR "Brunschwig operation"))))

AND

(((surgical OR free OR tissue OR adipofascial OR adipo-fascial OR "anterolateral thigh" OR "antero-lateral thigh" OR ALT OR "medial thigh" OR "posterior thigh" OR pudendal-thigh OR chimeric OR fasciocutaneous OR fascio-cutaneous OR gracilis OR IGAP OR I-GAP OR inguinal OR groin OR island OR muscle OR myocutaneous OR myo-cutaneous OR muscular-cutaneous OR musculocutaneous OR musculo-cutaneous OR perforator OR skin OR cutaneous OR dermal OR pedicle* OR bipedicle* OR transposition* OR Singapore OR VRAM OR gluteal OR gluteus OR omentum OR omental OR peritoneum OR peritoneal OR V-Y OR fillet OR local OR advancement OR "fasciae latae" OR Martius) NEAR/2 (flap OR flaps OR graft OR grafts)) OR "free inguinal graft" OR "skin pedicle" OR "flap repair" OR "flap reconstruction*" OR "V-Y plasty"))

NOT DT=(Meeting Abstract)

Search narrative

- The first two search concepts ("pelvic cancer" and "pelvic surgery") were duplicated and worked out in two different ways which, in turn, were combined with OR. In the first way, general cancer terms like "oncolog*" or "cancer" were included without restriction with an anatomical term of the pelvis. In the second way, general surgery terms such as "reconstruct*", "plastic surgery" or "microvascular surgery" were included without restriction with an anatomical term of the pelvis. The reason for this was that anatomical terms of the pelvis could be explicitly named by the authors only in connection with either cancer or surgery but not necessarily with both.
- Also, the proximity operator was replaced by AND in those "pelvic cancer" and "pelvic surgery" concepts that were not restrained by anatomical terms. For the "pelvic cancer" concept, this replacement with AND also involved the following remodeling: The nested ANDed brackets *((anatomy terms) AND (cancer terms))* with subject headings and text words were fused.

File S3. Overview of the PROM tools used and their respective frequency

No.	PROM	Full Name	Frequency
		**Generic PROMs (n=5)**	
**1**	SF-36	Short Form 36	1
**2**	BIS	Body Image Scale	1
**3**	EQ-5D	EQ-5D EuroQol Group, Rotterdam, The Netherlands) quality-of-life index	2
**4**	MOS SF-36	Medical Outcome Study Short Form 36	1
**5**	Cleveland Clinic QoL	Cleveland Clinic Quality of Life Questionnaire	1
		**General oncology PROMs (n=3)**	
**1**	EORTC QLQ-C30	European for Research and Treatment of Cancer Quality of Life	4

**2**	EORTC QLQ- CR29	European Organization for Research and Treatment of Cancer Quality of Life Questionnaire Colorectal Cancer	3
**3**	FACT-G	Functional Assessment of Cancer Therapy – General	1
		**Gynecological oncology-specific PROM (n=1)**	
**1**	FACT-V	Functional Assessment of Cancer Therapy – Vulva	1
	**PROMs relating to sexual function/pelvic floor function/incontinence (n=4)**		
**1**	FSFI	Female Sexual Function Index	4
**2**	FIQL	Fecal incontinence quality of life scale	1
**3**	mSAQ	Modified Sexual Adjustment Questionnaire	1
**4**	SVQ	Sexual function Vaginal changes Questionnaire	1
